# Detection of *Mycobacterium tuberculosis* and Rifampicin Resistance Using GeneXpert MTB/RIF Assay at Enat Hospital, Central Ethiopia

**DOI:** 10.1155/2022/1250404

**Published:** 2022-01-18

**Authors:** Sebsib Selfegna, Amir Alelign

**Affiliations:** ^1^Department of Biology, College of Natural and Computational Sciences, Debre Berhan University, P.O. Box 445, Debre Berhan, Ethiopia; ^2^Department of Biology, College of Natural and Computational Sciences, University of Gondar, P.O. Box 196, Gondar, Ethiopia

## Abstract

**Background:**

Tuberculosis remains to be a public health threat in Ethiopia. However, the use of ill diagnostic methods and the lack of enough epidemiological information in the country contributed to the diagnostic delay and development of anti-TB drug resistance. Therefore, the present study is aimed at assessing the prevalence of pulmonary TB (PTB) and the development of drug resistance using GeneXpert MTB/RIF assay in Merhabete district, Central Ethiopia.

**Methods:**

A cross-sectional, health facility-based study was conducted from December 2019 to June 2020. Bacteriological examination and GeneXpert molecular diagnostic methods were used for the detection of *M*. *tuberculosis* and rifampicin resistance (RR). Descriptive statistics and logistic regression analysis were used to determine the possible association of risk factors with the occurrence of PTB and RR. *P* values of <0.05 were considered statistically significant.

**Results:**

The overall prevalence rates of PTB and RR *M*. *tuberculosis* were 11.2% and 15.8%, respectively. The logistic regression analysis revealed that being in the age group of 49-64 years was significantly associated with the occurrence of TB (*P* = 0.01). The odds of HIV-positive and retreatment study participants to be infected by *M*. *tuberculosis* were much more than those of HIV-negative and newly treated cases, respectively (*P* < 0.05). However, none of the sociodemographic and clinical patient characteristics was significantly associated with the development of RR-TB (*P* > 0.05).

**Conclusion:**

In the present study, high prevalence rates of PTB and RR *M*. *tuberculosis* were observed. The findings, which were attributed to different risk factors, suggested an urgent need for appropriate intervention measures to reduce the transmission of PTB and the development of anti-TB drug resistance in the study area.

## 1. Introduction

Tuberculosis (TB) remains to be a global public health concern. The disease is caused by a bacterium known as *Mycobacterium tuberculosis* (*M*. *tuberculosis*) [1]. *M*. *tuberculosi*s most commonly attacks the lung, and this is called pulmonary tuberculosis (PTB). PTB becomes a chronic illness and causes extensive scarring in the upper lobes of the lung.

In 2019, an estimated 10.0 million people fell ill with TB. Men and women (aged ≥15 years) accounted for 56% and 32% of the people who developed TB in the same year, respectively, whereas children (aged <15 years) shared about 12% of the total TB-positive cases for that year. Among all those TB cases, 8.2% were coinfected with HIV [2].

Drug-resistant TB continues to be a public health crisis. In 2019, nearly half a million people developed rifampicin-resistant TB (RR-TB) globally, of which 78% had multidrug-resistant TB (MDR-TB). In the same year, about 3.3% of new TB cases and 17.7% of previously treated cases had MDR/RR-TB [2].

Ethiopia remains to be one of the high-TB burden countries with an estimated incidence rate of 164/100,000 population and with a death rate of 28/100,000 population [3]. The Amhara region continues to be one of the hot spot areas for the disease in the country. From 2014 to 2017, the average notification rate of all TB forms in the region was 107/100,000 population [4].

In Ethiopia, the routine TB diagnosis was mostly relayed on insensitive methods such as smear microscopy. Subsequently, the ill diagnosis methods create a diagnostic delay that hinders disease control, enhances transmission, and increases healthcare costs. However, the recent introduction of the GeneXpert TB diagnostic method (an automated real-time polymerase chain reaction assay) enhances TB control efforts in resource-limited settings such as Ethiopia. This method contributes to the rapid and simultaneous detection of *M*. *tuberculosis* and RR [5]. However, little has been done in this respect in most endemic parts of the country. Hence, the current study is aimed at determining the prevalence of PTB and RR-TB and associated risk factors using the GeneXpert molecular methods among TB-suspected individuals in Merhabete district, Amhara region, Central Ethiopia.

## 2. Materials and Methods

### 2.1. Description of the Study Area

The study was conducted in Merhabete district, North Shewa Zone, Amhara region, Central Ethiopia. The district is located 182 km from Addis Ababa, the capital city of Ethiopia. Merhabete is located about 747 km east of the regional seat, Bahir Dar. The total population of the district is 113,678, and 95.5% of them have based their life on subsistence farming [6]. Enat Hospital, where the present study was conducted, had 73 beds, of which 12 of them were allocated for TB treatment.

### 2.2. Study Design and Population

A health facility-based cross-sectional study was conducted from December 2019 to June 2020 on presumptive PTB individuals who visited and sought TB treatment in Enat Hospital. All tuberculosis-suspected patients who visited the health facility seeking TB treatment during the study period were considered the study population. All TB-suspected patients manifesting clinical symptoms of TB, including fever, chronic cough for more than 2 weeks, and night sweats, and willing to participate in the study were included, whereas TB patients who were under TB treatment prior to the present study, patients incapable of producing sputum, patients who were suspected for ETB, and those who were less than 16 years of age at the time of the study were excluded from the study.

### 2.3. Sample Size Determination

The sample size for the present study was determined using a population proportion with the specified absolute precision formula [7]: *n* = *Z*_1−∝/2_^2^*p*(1 − *p*)/*d*^2^, where *Z*_1−∝/2_ is the standard normal variant (at 5% type 1 error (*P* < 0.05) which is 1.96), *p* = 13% (0.13) is the expected TB prevalence from a previous similar study [8], and *d* is the absolute error or precision taken as 5% (0.05). Accordingly, by adding a 10% nonresponse rate, which is 17, the total sample size was determined to be 190.

## 3. Data Collection Tools and Procedures

### 3.1. Sociodemographic Data

A structured questionnaire was developed and used to collect data about the sociodemographic and clinical characteristics of the study subjects. The questionnaire was first developed in English and then translated into the local language, Amharic, for appropriateness and clarity.

### 3.2. Specimen Collection

On-spot morning sputum specimens were collected from presumptive TB patients in Enat Hospital. Health workers advised TB suspects to cough and clear the back of the throat and then to give a good cough to bring up sputum. Samples were collected in a well-ventilated area. The individuals were given Falcon tubes to collect about 3 ml of their own sputum samples. However, those samples less than 3 ml in volume were rejected. Wrong sample containers, containers with leakage and breakage, and incomplete questionnaires were also rejected. Specimens were rechecked for fulfilling criteria and were placed in cold boxes or in the refrigerator until they were processed immediately.

### 3.3. Laboratory Methods by GeneXpert

All sputum samples were subjected to the GeneXpert MTB/RIF system (Cepheid, USA) in Enat Hospital's laboratory department. For GeneXpert MTB/RIF assay, about 0.5 ml of the sputum sediment which was resuspended in phosphate-buffered saline was treated with the sample reagent (1.5 ml). The mixture was then shaken by hand according to test instructions. The mixture was vortexed for 30 s to ensure all bacteria were resuspended. The sample was incubated for 15 min at 20-30°C, as per the manufacturer's instructions, with intermittent manual shaking [9]. The solution was then transferred to the Xpert cartridge using a Pasteur pipette, and the cartridge was loaded onto the Xpert machine for analysis. The results were visualized and printable in the *view results* window. Results from the Xpert MTB/RIF assay indicate whether or not MTB was detected in the samples. In some instances, the result was “invalid,” whereby the test was repeated. If MTB is detected, the results were stated whether resistance to rifampicin (RIF) was detected, not detected, or indeterminate.

## 4. Data Analysis and Interpretation

Statistical analysis was performed using SPSS software version 20. Descriptive statistics such as frequencies and percentages were used to characterize the study participants. The results of the study were also presented by using tables to depict the sociodemographic and clinical variables. Logistic regression analysis was used to determine the possible association of risk factors with the occurrence of PTB and RR *M*. *tuberculosis*. *P* value of <0.05 was considered a statistically significant association.

## 5. Results

### 5.1. Sociodemographic and Clinical Characteristics of TB Patients

In the present study, 170 presumptive TB patients participated with a response rate of 89.5%. The mean age of the participants was 45.7 years, and most of them, 53 (31.0%), were in the age range of 49-64 years. Males were more frequent, 110 (64.3%), than females. About 30% (51/170) of the respondents had no formal education. Seventy-three of the respondents (42.9%) lived in overcrowded urban settings, and nearly 47.1% of the participants had a family size of 6 and above individuals (Table 1).

Fifty-eight (34.1%) of the respondents were HIV positive. Thirty-six from one hundred seventy (21.2%) had TB treatment history who had started to take isoniazid (INH) prophylaxis. Of this, 26 (15.3%) were cured completely, 7 (4.1%) were failures, and 3 (1.8%) were defaulters due to medicine side effects and distance from health facilities. However, the majority of TB patients, 134 (78.8%), were newly diagnosed cases (Table 1).

### 5.2. Prevalence of Pulmonary TB

In the present study, the overall prevalence of PTB was 11.2% (19/170). About 89.5% (17/19) of the TB-positive cases were from previously treated ones. The proportion of *M*. *tuberculosis* was 12 (10.9%) in males and 7 (11.9%) in females. *M*. *tuberculosis* was detected most frequently in the age group of 16-32 years, 9 (21.4%), than the other age groups; however, only a single TB case was detected among respondents of 65 or above years of age. *M*. *tuberculosis* was detected in about 94.4% of the retreatment cases, whereas only 1.3% of the newly diagnosed cases were positive for *M*. *tuberculosis*. Twelve out of nineteen (63.2%) TB cases were HIV positive. Most of the confirmed TB cases (13/19) had been clinically characterized as having either fever or chest pain (Table 2).

The logistic regression analysis revealed that being in the age group of 49-64 years was significantly associated with the occurrence of TB (*P* = 0.01). The odds of HIV-positive and retreatment study participants to be infected by *M*. *tuberculosis* were much more than those of HIV-negative and newly treated cases, respectively. These associations were also statistically significant (*P* < 0.05) (Table 2).

### 5.3. Rifampicin-Resistant TB

Of the 19 confirmed PTB cases, 3 (15.8%) were resistant to rifampicin. The proportion of RR *M*. *tuberculosis* was 2 (18.2%) and 1 (16.7%) among males and females, respectively. From the different age groups, no RR-TB was detected in those 49 and above years of age. Two (20.0%) RR-TB cases were detected among patients with PTB/HIV coinfection. All the RR-TB cases were from previously treated patients and those who had a family size of 1-3 individuals (Table 3). The logistic regression analysis revealed that none of the sociodemographic and clinical patient characteristics were significantly associated with the development of RR-TB (Table 3).

## 6. Discussion

Our study revealed that the overall prevalence of TB in the study area was 11.2%. However, this finding is observed to be lower than the previous studies conducted in the Metehara sugar factory, Eastern Ethiopia (14.2%) [10], University of Gondar Hospital (24.6%) [11], Debre Berhan Referral Hospital (13%) [8], and Tigray region (24.3%) [12]. Earlier studies in some parts of Africa and elsewhere in the world also reported higher prevalence rates of TB: Kenya (39.2%) [13], Nigeria (22.9%) [14], and India (32.9%) [15]. The low prevalence of TB in the current study was due to the fact that we have included presumptive TB cases, unlike the other studies, which included confirmed TB cases. Conversely, the present TB prevalence was found to be higher than other previous studies conducted in the region such as Debre Berhan and Dessie (2.6%) [16], South Gondar Zone (6.3%) [17], and other parts of Africa such as Eritrea (7.8%) [18]. In another study conducted by Mulatu and others [19], the prevalence of TB among those in prison at Benishangul Gumuz Region was 0.24%.

This discrepancy in the prevalence of TB could be a result of the difference in methodological approaches (smear microscopy, culture, and GeneXpert), community, and geographical locations and due to the study population difference and duration of the study. The lower sample size used in the present study was also the possible reason for the high prevalence of TB than the above studies.

In the present study, TB-HIV coinfection was found to be high (20.7%) which was in line with previous studies conducted across the country (20.3%-24.3%) [10, 20–23]. On the contrary, the current finding was found to be lower as compared to other previous studies conducted in Tigray (33.3%) [11], another part of the Amhara region (27.7%) [24]. Conversely, the present finding was observed to be higher than those conducted in other regions such as Tigray (12.1%) [25], another part of Ethiopia (17.2%) [26], and India (11.9) [15]. The possible explanation for this variation could be differences in methodological approaches and implications of possible policy direction in the diagnostic approaches of TB-HIV coinfected patients.

In the current study, MTB was detected in all age groups included in the study. A significantly (*P* < 0.05) higher prevalence of TB was observed in the age group 16-33 years than those above 33 years old. This was in agreement with previous studies conducted in northeast Ethiopia in which all TB cases were found in the young age group (17–44 years) of the participants [8, 16]. This could be due to the higher chance of exposure to the infection because of higher social interaction and mobility by the young age groups than the older ones. This could be also an implication of the impact of the disease on the productivity of economically active population groups, leading to socioeconomic consequences in the study area in particular.

In the current study, previous TB treatment was observed to associate significantly with MTB infection. This finding was supported by previous studies conducted by Fite and others [26]. The higher prevalence of MTB in the retreatment cases implicated a need for due attention in designing appropriate strategies in the treatment and control of the disease in these target groups of the population, as this could lead to high TB transmission to the new cases and possible development of drug-resistant TB in the study area.

On the other hand, about 15.8% of the confirmed TB cases were identified to be resistant to rifampicin (RR). The prevalence of RR-TB in this study was supported by previous studies in Gondar (15.8%) [11] and Nigeria (14.7%) [14]. The present finding was higher than previous studies conducted in other parts of Africa such as Kenya (3.7%) [27] and Zambia (5.9%) [28]. Lower prevalence of RR-TB was also observed in other parts of Ethiopia such as Adigrat General Hospital (9.1%) [12], Debre Markos Referral Hospital (10.3%) [29], Felege Hiwot Referral Hospital and Debre Tabor Hospital (9.3%) [30], Addis Ababa (9.9%) [31], and elsewhere (10.5%) [32]. However, our finding was lower than previous studies conducted in South Gondar (18.0%) [33], Congo (42.2%) [34], and China (17.6%) [35]. The possible explanation for the variations in RR-TB could be due to differences in sample size, the method of diagnosis, and population types, as the higher prevalence of the above studies as compared to ours was attributed due to the drug-resistant suspected populations, unlike the presumptive TB patients included in the current study.

Despite the observed variation in the proportions of RR-TB in our study, none of the sociodemographic or clinical characteristics were found to be associated with anti-TB drug resistance. This observation is supported by other studies in Ethiopia [33, 36] and Zambia [28]. In this study, two of the three RR-TB cases were contributed by younger age groups of 16-32 years which was in agreement with the previous finding in South Gondar by Alelign and others. The authors reported that younger adults between the ages 18-30 contributed 45% of drug-resistant TB cases [32]. All the RR-TB cases in the present study were retreatment cases, and such findings were also reported by previous studies [33, 36]. This was due to the fact that the previously treated cases had possible interruptions in their course of anti-TB treatment which might predispose the development of drug-resistant *M*. *tuberculosis* strains circulating and causing TB in the study area.

## 7. Conclusion

In the present study, the overall prevalence of pulmonary tuberculosis and rifampicin-resistant *M*. *tuberculosis* was found to be high. HIV coinfection and retreatment of the cases were some of the major risk factors for the occurrence of the disease. Hence, maximizing efforts for early detection of anti-TB drug resistance and controlling the occurrence of the disease are needed to reduce the public health impact of the disease in the study area.

## Figures and Tables

**Table 1:** Sociodemographic and clinical characteristics of study participants at Enat Hospital, Merhabete district, Central Ethiopia, 2020 (*N* = 170).

Variables	Frequencies	Percentages
Age (years)		
16-32	44	25.7
33-48	44	25.7
49-64	53	31.0
≥65	29	17.0
Sex		
Male	110	64.3
Female	60	35.1
Level of education		
Formal education	119	70
Nonformal education	51	30.0
Family size		
1-3	32	18.8
4-5	58	34.1
≥6	80	47.1
Residence area		
Rural	97	57.1
Urban	73	42.9
Patient HIV status		
HIV positive	58	34.1
HIV negative	112	65.9
TB treatment history		
Retreatment	36	21.2
New cases	134	78.8
TB treatment outcome (*n* = 36)		
Cured	26	15.3
Failure	7	4.1
Defaulter	3	1.8

**Table 2:** Association of study participant characteristics to the prevalence of TB at Enat Hospital, Merhabete district, Central Ethiopia, 2020 (*N* = 170).

Variables	*M*. *tuberculosis* result by GeneXpert™		Total (%) (*N* = 170)	AOR (95% CI)	*P* value
	Detected (%) (*n* = 19)	Not detected (%) (*n* = 151)			
Age (years)					
16-32	9 (20.4)	33 (78.6)	42 (24.7)	1.00	
33-48	7 (15.9)	37 (84.1)	44 (25.9)	0.69 (0.23-2.07)	0.51
49-64	2 (3.7)	52 (96.3)	54 (31.8)	0.14 (0.02-0.69)	0.01
≥65	1 (3.3)	29 (96.7)	30 (17.6)	0.12 (0.01-1.05)	0.06
Sex					
Male	12 (10.9)	98 (89.1)	110 (64.3)	1.00	
Female	7 (11.7)	53 (88.3)	60 (35.1)	1.07 (0.40-2.90)	0.88
Family size					
1-3	6 (18.8)	26 (81.2)	32 (18.8)	1.00	
4-5	6 (10.3)	52 (89.7)	58 (34.1)	0.50 (0.14-1.70)	0.26
≥6	7 (8.8)	73 (91.2)	80 (47.1)	0.41 (0.12-1.35)	0.14
Residence area					
Rural	11 (11.3)	86 (88.7)	97 (57.1)	1.00	
Urban	8 (11.0)	65 (89.0)	73 (42.9)	0.96 (0.36-2.52)	0.93
Patient HIV status					
HIV positive	12 (20.7)	46 (79.3)	58 (34.1)	1.00	
HIV negative	7 (6.7)	98 (93.3)	105 (65.9)	0.27 (0.10-0.74)	0.01
TB treatment history					
Retreatment	17 (94.4)	1 (5.6)	18 (21.2)	1.00	
New cases	2 (1.3)	150 (98.7)	152 (78.8)	0.001 (0.00-0.01)	<0.0001
Symptoms					
Fever	6 (19.4)	25 (80.6)	31 (18.2)	1.00	
Chest pain	7 (16.7)	35 (83.3)	42 (24.7)	0.83 (0.24-2.78)	0.76
Loss of appetite	2 (7.4)	25 (92.6)	27 (15.9)	0.33 (0.06-1.81)	0.20
Weight loss	3 (9.4)	29 (90.6)	32 (18.8)	0.43 (0.09-1.90)	0.26
Shortness of birth	1 (3.7)	26 (96.3)	27 (15.9)	0.16 (0.01-1.42)	0.10
Bloody mucus with coughing	0 (0)	11 (100)	11 (6.5)	0.17 (0.01-3.29)	0.24
Total	19 (11.2)	151 (88.2)	170 (100)		

**Table 3:** Association of patient characteristics to the pattern of RIF at Enat Hospital, Merhabete district, Central Ethiopia, 2020.

Variables	Pattern of RIF		Total (%) (*N* = 17)^X^	AOR (95% CI)	*P* value
	Sensitive (%) (*n* = 14)	Resistant (%) (*n* = 3)			
Age (years)					
16-32	7 (77.8)	2 (22.2)	9 (52.9)	1.00	
33-48	4 (80.0)	1 (20.0)	5 (29.4)	0.87 (0.05-12.97)	0.92
49-64	2 (100)	0 (0)	2 (11.8)	0.60 (0.02-17.2)	0.76
≥65	1 (100)	0 (0)	1 (5.9)	1.00 (0.03-33.3)	1.00
Sex					
Male	9 (81.8)	2 (18.2)	11 (64.7)	1.00	
Female	5 (83.3)	1 (16.7)	6 (35.3)	0.90 (0.06-12.58)	0.94
Family size					
1-3	3 (50.0)	3 (50.0)	6 (35.3)	1.00	
4-5	5 (100)	0 (0)	5 (29.4)	0.09 (0.00-2.34)	0.15
≥6	6 (100)	0 (0)	6 (35.3)	0.07 (0.00-1.95)	0.12
Residence area					
Rural	7 (77.8)	2 (22.2)	9 (52.9)	1.00	
Urban	7 (87.5)	1 (12.5)	8 (47.1)	0.50 (0.03-6.86)	0.60
TB treatment history					
Retreatment	13 (81.3)	3 (18.7)	16 (94.1)	7.00 (0.16-291.3)	0.30
New cases	1 (100)	0 (0)	1 (5.9)	1.00	
Patient HIV status					
HIV positive	8 (80.0)	2 (20.0)	10 (58.8)	1.50 (0.10-20.6)	0.76
HIV negative	6 (85.7)	1 (14.3)	7 (41.2)	1.00	
Total	14 (82.4)	3 (17.6)	17 (100)		

^X^Two *Mycobacterium tuberculosis* samples were RIF indeterminate, hence excluded from the analysis. AOR: adjusted odds ratio; CI: confidence interval; RIF: rifampicin.

## Data Availability

The datasets generated and/or analyzed during the current study are available from the corresponding author on reasonable request.

## Abbreviations

AOR: Adjusted odds ratio
CI: Confidence interval
HIV: Human immunodeficiency virus
MDR: Multidrug resistance
MTB: *Mycobacterium tuberculosis*
PTB: Pulmonary tuberculosis
RIF: Rifampicin
RR: Rifampicin resistance
SPSS: Statistical Package for Social Sciences
TB: Tuberculosis.

## Additional Points

*Limitations*. As our study was conducted in a resource-limited setting and due to a lack of trained pathologists, we did not include extrapulmonary TB (TB other than the lung) cases. Sociodemographic and clinical patient variables included were also limited.
